# A Pipelined Tracer-Aware Approach for Lesion Segmentation in Breast DCE-MRI

**DOI:** 10.3390/jimaging7120276

**Published:** 2021-12-14

**Authors:** Antonio Galli, Stefano Marrone, Gabriele Piantadosi, Mario Sansone, Carlo Sansone

**Affiliations:** 1 Department of Electrical Engineering and Information Technology (DIETI), University of Naples Federico II, Via Claudio 21, 80125 Naples, Italy; antonio.galli@unina.it (A.G.); mario.sansone@unina.it (M.S.); carlo.sansone@unina.it (C.S.); 2 Altran Italia S.p.A., Centro Direzionale, Via Giovanni Porzio, 4, 80143 Naples, Italy; gabriele.piantadosi@altran.it

**Keywords:** breast, DCE-MRI, eras/epochs, lesion segmentation, UNet, 3TP

## Abstract

The recent spread of Deep Learning (DL) in medical imaging is pushing researchers to explore its suitability for lesion segmentation in Dynamic Contrast-Enhanced Magnetic-Resonance Imaging (DCE-MRI), a complementary imaging procedure increasingly used in breast-cancer analysis. Despite some promising proposed solutions, we argue that a “naive” use of DL may have limited effectiveness as the presence of a contrast agent results in the acquisition of multimodal 4D images requiring thorough processing before training a DL model. We thus propose a pipelined approach where each stage is intended to deal with or to leverage a peculiar characteristic of breast DCE-MRI data: the use of a breast-masking pre-processing to remove non-breast tissues; the use of Three-Time-Points (3TP) slices to effectively highlight contrast agent time course; the application of a motion-correction technique to deal with patient involuntary movements; the leverage of a modified U-Net architecture tailored on the problem; and the introduction of a new “Eras/Epochs” training strategy to handle the unbalanced dataset while performing a strong data augmentation. We compared our pipelined solution against some literature works. The results show that our approach outperforms the competitors by a large margin (+9.13% over our previous solution) while also showing a higher generalization ability.

## 1. Introduction

World Cancer Research Fund reports [1] indicate breast cancer as the most common among women, with about 25% of all cancer occurrences. Still today, early diagnosis represents a key factor for reducing death rates since breast cancer usually develops and spreads unhindered, showing symptoms only in advanced stages [2]. The World Health Organization (WHO) recommends mammography as breast cancer’s main screening methodology [3], thanks to its high resolution and detection ability. Nonetheless, in the last years, researchers are focusing on the use of other imaging techniques, mostly because of (i) mammography’s non-suitability for under-forty women, since density may lead to over-diagnosis, and (ii) because of the use of ionising radiations. Among all medical imaging techniques, Dynamic Contrast-Enhanced Magnetic-Resonance Imaging (DCE-MRI) is showing promising results for the early detection of different types of tumours [4], proving particularly suitable for breast-cancer detection in women with extremely dense breast tissue [5]. DCE-MRI leverages a contrast agent (CA) to highlight the tissues’ vascularisation physiological and morphological characteristics [6]. In particular, since the CA is a (super)paramagnetic liquid characterised by specific absorption and release times, its spread at different speeds highlights lesions over healthy tissues.

**Figure 1:**
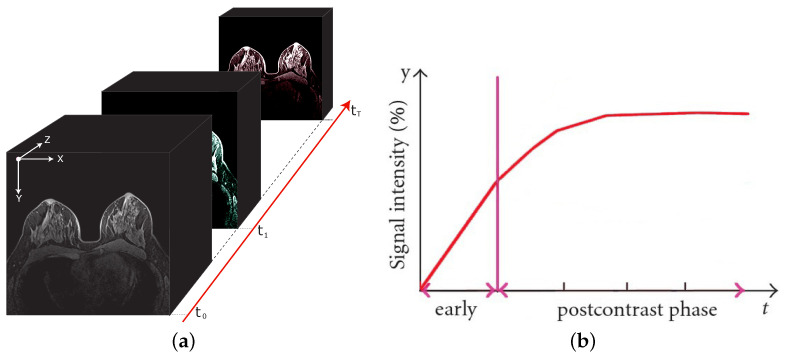
A breast DCE-MRI study and an illustrative time intensity curve. On the left (**a**), the 4D multimodal volume, with the signal intensity variations reflecting the contrast agent flow over time. On the right (**b**), the corresponding (illustrative) time intensity curve for a single voxel. The vertical line separates the pre-contrast (early) from post-contrast injection instants.

A typical DCE-MRI study requires the scan of multiple (at least two) 3D volumes, before (pre-contrast) and after (post-contrast) the CA intravenous injection. Therefore, a DCE-MRI scan can be considered as a 4D multimodal volume (Figure 1a) having three (x,y,z) spatial and a single (t) temporal dimensions. As a result, each DCE-MRI voxel (an over-time 3D pixel) is associated with a time intensity curve (TIC—Figure 1b) reflecting the absorption and the release dynamic of the CA, as a function of the tissue-vascularisation characteristics [6]. 

If, on the one hand, the DCE-MR imaging procedure is proving to be effective in tumour diagnosis, the flip side of the coin is in the huge amount of produced data and in its long acquisition times. While the former might only impact the processing time, the latter is more problematic since it can introduce motion artefacts due to inevitable patient movements. These drawbacks can make the DCE-MRI data tough to analyse without the support of a computer-aided detection (CAD) system aimed at supporting the physician in the inspection of biomedical images. CAD systems consist of several modules, each intended to perform a given task. In the case of breast DCE-MRI, one of the hardest tasks is the *lesion segmentation*, namely, the pixel-wise identification of a suspected region of interest (ROI) [7]. Indeed, with the spread of high-precision tasks, such as MRI-guided robotic surgery [8], neoadjuvant chemoradiation [9], etc., the coarse lesion detection is no longer sufficient (see Section 2).

As with many other computer vision tasks, several CAD systems make use of machine learning to provide automatic breast lesion segmentation. Moreover, with the spread of Deep Learning (DL), new studies have recently been conducted to explore its applicability to this task, intending to exploit their effectiveness of autonomously learning a suitable set of features for the task under analysis. Despite some works showing promising results [10], we argue that solutions based on a “naive” use of DL might have limited effectiveness as the presence of a contrast agent results in the acquisition of multimodal 4D images requiring thorough processing before training a DL model. Indeed, we strongly believe that the intrinsic physiological characteristics of DCE-MRI data should be considered to design a method able to properly exploit all the available information. With this aim in mind, we thus propose a pipelined approach designed to:

- leverage the Three-Time-Points (3TP) [11] method to take into account for the contrast agent course without imposing hard constraints on the total number of acquired post-contrast series;
- make use of a motion-correction Technique tMCT) to deal with patient involuntary movements;
- exploit an innovative training schema, introduced for the first time in this study, which was conceived to perform data augmentation and class balancing in the contest of medical data while acting as training regularization;
- perform the lesion-segmentation task by means of a modified U-shaped CNN [12].

With respect to our previous work [13], where we first introduced the idea of 3TP slices for lesion segmentation in breast DCE-MRI, here we present a more-complete solution that also takes into account motion artefacts, data balancing, and augmentation. The resulting pipeline, as described in Section 4, allows us to increase both the segmentation effectiveness and the generalization ability, outperforming all the considered competitors by a large margin. The article continues as follows: Section 2 concisely explores the lesion-segmentation literature; Section 3 describes the proposed 3TP U-Net approach; Section 4 reports the obtained results, comparing them against some state-of-the-art competitors; finally, Section 5 discusses the obtained findings and provides some conclusions.

## 2. Related Works

**Figure 2:**
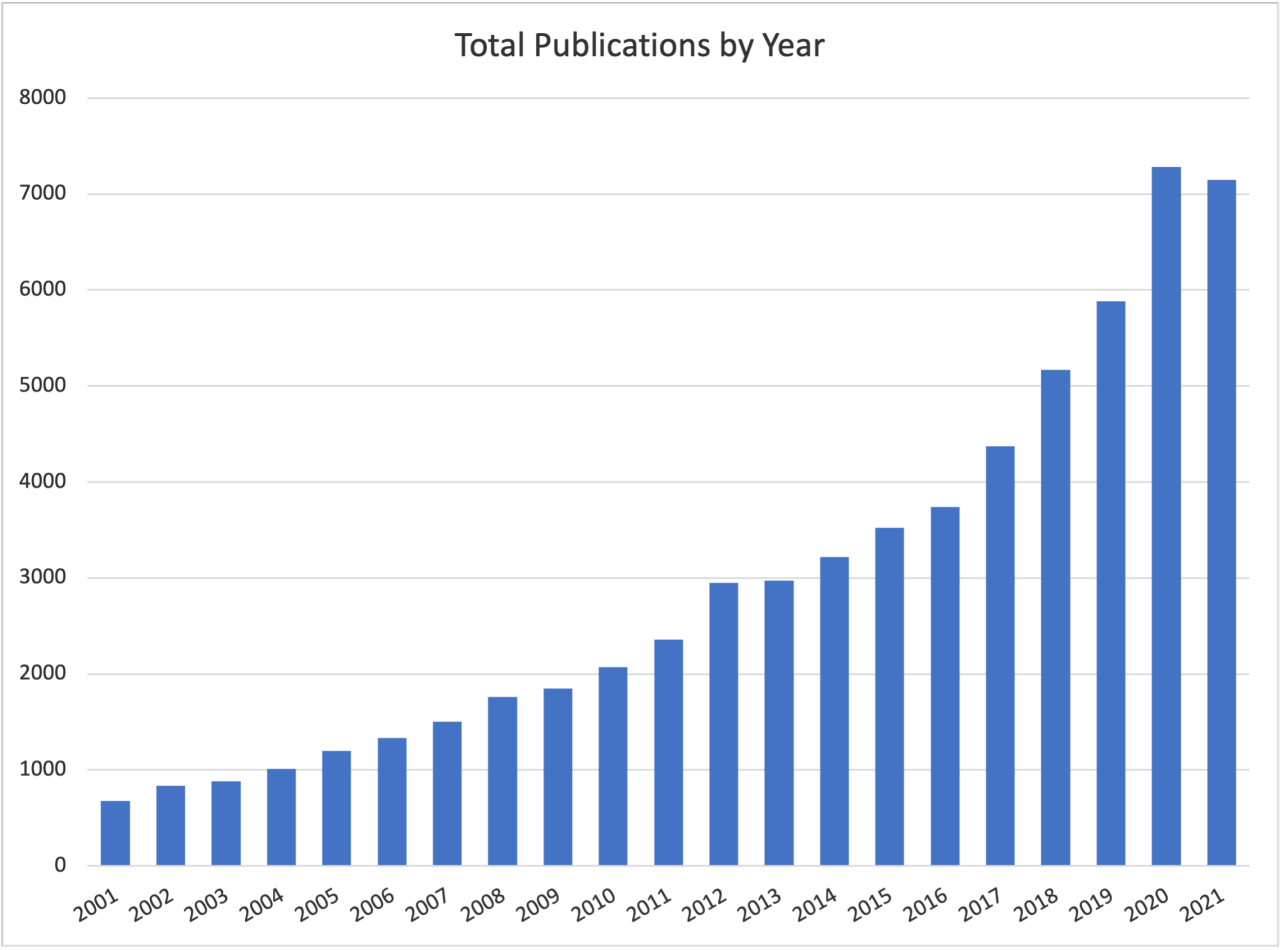
Number of studies published between 2001 and the first ten months of 2021 on Google Scholar filtered by “Breast Lesion Segmentation” as topic keyword.

With the aim of better framing our work, we briefly provide a summary of the most-cited DCE-MRI automatic breast lesion-segmentation approaches so far proposed. The lesion-segmentation task consists in classifying each voxel as belonging or not to a lesion, generating a binary mask whose function is to precisely identify the lesion. The result is a region of interest (ROI) delimiting a tumour lesion (i.e., a portion of tissue) that should be further investigated to determine its aggressiveness. Lesion segmentation is thus different from lesion detection, whose aim is to simply identify the rough portion of tissues (e.g., with a bounding box) potentially affected by a lesion. Over the years, researchers are more and more focusing on the segmentation task (Figure 2), mostly as a result of the need for precise lesion localization as input to automatic lesion-ablation procedures.

Literature approaches can be grouped according to different characteristics. With the rise of deep learning, one of the most intuitive is to separate non-deep- from deep0based approaches: the former rely on features “hand-crafted” by domain experts to describe the ROI characteristics, while the latter rely on the ability of deep neural networks to autonomously learn the set of features that better suit the task under analysis. Focusing on non-deep approaches, these can be further grouped into three sets (Table 1) based on the characteristics of the lesion they look for.

In all cases, once the lesion has been described using one or more sets of features, the actual lesion segmentation is performed by means of a pattern-recognition method. Three are the most-used strategies: a filtering-based (FILT) method, making use of a simple threshold to separate healthy from lesioned tissues; a morphological-based (MORPH) method, using geometric iterative algorithms such as Region Growing, Graph-Cut, or Active Contour; and a model-based (MODEL) method, exploiting machine-learning techniques. Table 2 summarizes some among the most-cited (over the last 10 years) non-deep literature proposals, reporting for each: the year of publication, whether it uses a motion-correction technique (MCT) to deal with motion artefacts, the used feature set, the approach category, and the obtained performance as reported by the authors.

Moving to deep-based approaches, it is worth noting that the wider part of the literature focuses on lesion detection, mostly as a result of the success of deep learning in object-detection tasks. However, more recently some authors started proposing approaches for the DCE-MRI breast lesion segmentation:

- In [31], the authors proposed a solution based on the stacking of three parallel ConvLSTM [32] networks to extract temporal and 3D features, followed by a four-layer fully convolutional U-Net;
- In [33], noting that the lesion-segmentation task can be treated as a classical semantic segmentation (i.e., dividing the input image into regions of interest), the authors explored the suitability of the well-known U-Net and SegNet deep-semantic-segmentation networks;
- finally, in [34], the authors conducted a task-based assessment on the effectiveness of convolutional neural networks (CNNs) in emulating segmentation made by experienced radiologists.

Despite some works showing interesting solutions (such as the use of ConvLSTM layers), none of them either leverages the contrast agent course in terms of seconds after its injection or takes into account motion artefacts, data augmentation, and class balancing. This results in approaches that are harder to reproduce on datasets different from those the procedure has been designed for. It is also likely that these approaches will tend to generalize worse on other datasets.

## 3. Proposed Approach

**Figure 3:**
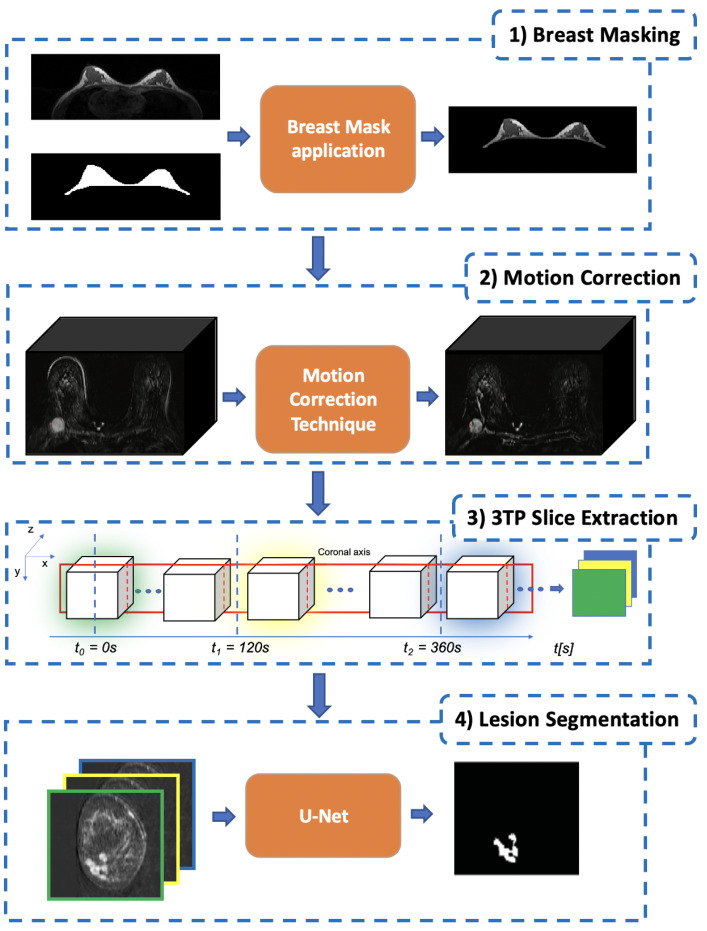
The proposed pipelined segmentation schema: in the first stage, all the extraneous tissues and background air are removed; in the second stage, a motion-correction technique is used to register each post-contrast 3D-volume to the pre-contrast one; in the third stage, for each slice, the corresponding 3TP slice (a three-channel image) is generated by concatenating homologous slices identified by the three-time points defined in [11]; finally, in the fourth stage, each lesion is segmented by using a modified U-Net to produce the final lesion binary mask.

As described in the previous sections, lesion segmentation is a major task that needs to take into account several aspects to result in effective and reproducible outcomes. To this aim, in in this work, we introduce a multi-staged DL-based methodology consisting of a series of steps each intended to address a particular aspect (Figure 3):

1. The first stage is **Breast Masking** (Section 3.1), in which the extraneous tissues (muscles, bones, air background, etc.) are removed from the acquired volume;
2. Once the volume contains only breast voxels, the successive step is to perform **Motion Correction** (Section 3.2) to reduce the noise (i.e., misalignment between the same slice across different temporal acquisitions) introduced by involuntary patient movements;
3. The third stage is the **3TP Slice Extraction** (Section 3.3), a procedure intended to standardise the input data number of channels regardless of the number of acquired pre and post-contrast series [13]. To do so, each over-time slice (i.e., the set of all the same slices extracted from the acquired series) is transformed into a three-channel image by stacking the three instances acquired at very specific time points (expressed in seconds after the CA injection) as suggested in [11], making the approach suitable for different DCE-MRI acquisition protocols;
4. The final stage is the ***Lesion Segmentation*** (Section 3.4), in which each lesion is segmented and the corresponding binary mask generated. Among all DL approaches, we focused on a U-Shaped Convolutional Neural Network (U-Net) [12] for its characteristic to autonomously learn the best mapping between the image input and the segmentation-mask output.

Besides the pipelined approach, the other key aspect of this work is the proposed **“Eras/Epochs” Training Schema** (Section 3.5), a novel data-feeding procedure explicitly designed to perform a suitable data augmentation while supporting class balancing and training regularization.

### 3.1. Breast Masking

DCE-MRI scanners acquire data not only from the tissues under analysis (e.g., the breast) but also from surrounding ones (e.g., pectoral muscle) as well as from the background (i.e., the air). This results in a huge amount of data that impacts the required computational effort and threatens to introduce information that is not actually useful for the lesion segmentation. Breast masking (BM) is thus the stage aimed in facing these problems, by generating a binary mask that includes only the breast parenchyma, while removing all the extraneous tissues (pectoral muscle, chest, etc.) and background air. The result of this stage is a 4D volume in which all the voxels not referring to breast tissues are set to a signal intensity value of 0. In this work, we rely on our fully automated breast-mask-extraction algorithm [35] based on a multi-planar 2D U-net. The core idea is to leverage three different U-Nets to extract the breast mask along each anatomical plane and then obtain a single breast mask by using a weighted voxel-level combining strategy. The result is a very general solution able to work on different datasets with a high median segmentation accuracy (>98%) and a neoplastic lesion coverage of 100%.

### 3.2. Motion Correction

Although DCE-MRI has been demonstrating great effectiveness in the screening of tumours, one of its drawbacks is in the long acquisition time (usually tens of minutes), which requires the patient to remain as steady as possible. For this reason, even physiological movements (e.g., breathing) may introduce motion artefacts that could negatively affect the automatic analysis of DCE-MRI data.

As a consequence, motion-correction techniques (MCT) have attracted a great deal of attention in the breast, as well as in other organs, DCE-MRI [36,37]. The goal of an MCT is to re-align (register) each voxel in the post-contrast series to the corresponding one in the pre-contrast image. MCTs can be grouped based on the type of transformation used to re-align two images. Several approaches have been so far proposed [38,39], and, although most of them were designed for natural images, more recently, some have been modified to be used with biomedical images [40,41]. The main reason why we consider a motion-correction stage is because, in previous work, we showed that even deep neural networks can benefit from it [42]. Unfortunately, choosing the most-appropriate MCT is not straightforward since we proved that there is not a single motion-correction technique always performing better than the others when applied to distinct patients or to distinct DCE-MRI protocols [43]. Nonetheless, and even though the proposed approach can be used with any MCT, in this work, we make use of a 3D non-rigid intensity-based registration provided by Elastix [41], an open-source software collecting image-registration techniques for medical images, as it showed to be among the most effective [7].

### 3.3. 3TP Slice Extraction

As introduced in Section 1, a DCE-MRI study involves the acquisition of several 3D volumes over time, resulting in a 4D structure having three spatial (*x, y* and *z*) and one temporal (*t*) dimension. The number of acquired 3D volumes depends on the number of temporal acquisitions *t* (pre/post-contrast series). This is a crucial aspect since the number of acquired post-contrast series determines the number of post-contrast replicas for each pre-contrast slice. Since across the different acquired volumes, slices in the same position refer to the same portion of the patient’s body, each slice can be interpreted as a multi-channel image, where the number of channels is equal to the number of acquired volumes (both pre and post-contrast). This multi-channel image represents the temporal evolution of the tissues comprised within the slice boundaries, during the contrast agent flowing. Despite this making DCE-MRI slices extremely rich in information, the design of machine-learning algorithms able to exploit this temporal structure is not straightforward. Indeed, not only the number of acquired post-contrast series but also the time interval between different series can strongly vary across the acquisition protocols of different medical centres. This is a crucial aspect to address when designing a method intended to leverage the temporal characteristics of DCE-MRI.

To make the proposed approach more general, in a previous work [13], we proposed to not directly use those multi-channel images. Instead, we proposed to leverage the 3TP method [11] to identify the most-meaningful temporal acquisitions from which the slices to be used could be extracted to generate the multi-channel image that will be fed to the deep-learning model. In particular, in [11], the authors showed that breast-lesion analysis can be successfully performed by only focusing on three temporal acquisitions (here named 3TP) *uniquely identified in terms of seconds after the CA injection*: pre-contrast ($t_{0}$); 2 min after ($t_{1}$); 6 min after ($t_{2}$). Leveraging the 3TP idea, for all the slices, we generated the corresponding 3TP image whose three channels consist of the same slice extracted from the volumes acquired at the time instances nearest to $t_{0}$, $t_{1}$, and $t_{2}$ (third block in Figure 3). The selection of these specific three time points makes the proposed approach more general, allowing to always feed the network with a three-channel image (regardless of the number of acquired post-contrast series) able to effectively synthesise the contrast agent course while reducing the required computational effort. It is worth noting that DCE-MRI voxels usually show a high anisotropy. Therefore, to maximise the informative content, this process is executed considering slices extracted over the projection having the higher spatial resolution (i.e., one among [x,y], [x,z], or [y,z]).

### 3.4. Lesion Segmentation

To perform the lesion segmentation, we propose to use a U-Shaped CNN [12] trained on the 3TP images. The network is a multi-level architecture, having an encoder (capturing the context) and a decoder (realising the precise segmentation) side. Compared to the original architecture, intended for microscopic images, we introduced [13] some changes:

1. We set the output feature-map to a single channel (and not one for each class as in the standard U-Net), with the aim of both helping the training convergence and to obtain a single probability prediction associated with each voxel. This comes at the cost of the need for a thresholding operation to obtain the desired binary segmentation map from the probabilistic output;
2. Since breast DCE-MRI images do not have breast tissues on the borders, we preferred to preserve the output shape by using a zero-padding with a size-preserving strategy;
3. We introduced a batch-normalization [44] stage after each rectified linear unit (ReLU) activation function block, to take into account the wide inter/intra patient variability.

**Figure 4:**
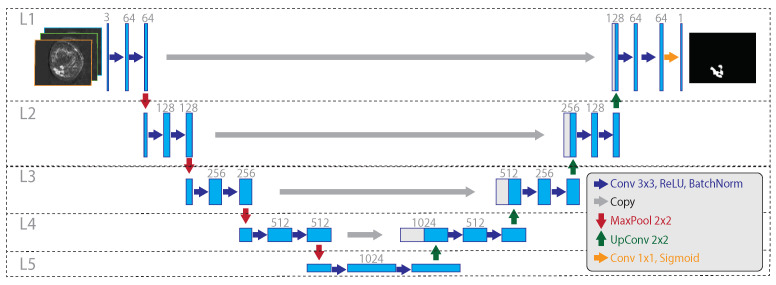
The considered 3TP U-Net architecture. On the left, the encoding side gradually decreases the spatial resolution while increasing the feature size. On the right, the decoding side gradually increases the spatial resolution from the inner embedding to the final output mask. Dotted lines and capital L highlight the network levels, with L5 being the deeper one. Big grey arrows illustrate the sharing of the cropped feature map, within the same layer, from the encoding to the decoding side, happening during the up-sampling. Compared to the classical U-Net architecture, the considered model varies for the use of batch normalization after each ReLU activation (in both encoding and decoding sides), for the use of zero padding, and for the use of a single output map.

The resulting network (Figure 4) has two multi-level sides, both consisting in the repeated sequence of some functional blocks: the encoding side uses 2D convolution (3 × 3 kernel, zero-padding, stride 1 × 1) followed by ReLU activation, batch normalization, and max-pooling (stride 2 × 2); the decoding side uses 2D up-convolution (2 × 2 kernel, zero-padding, stride 1 × 1) concatenated with the cropped feature map from the corresponding level on the encoding side, followed by 2D convolution (3 × 3 kernel, zero-padding, stride 1 × 1), ReLU activation, and batch normalization. In the deeper level, a 2D convolution (1 × 1 kernel, zero-padding, stride 1 × 1) is used to map each of the 64 component feature vectors to the network output. Finally, a probabilistic output is obtained by using the sigmoid activation function. The U-Net model was trained by using a segmentation-specific loss

(1) $$\mathrm{Loss}=1-\mathrm{DSC}\left(y_{\mathrm{net}},y_{\mathrm{gt}}\right),\;\mathrm{DSC}=2\times \frac{n(y_{\mathrm{gt}}\cap y_{\mathrm{net}})}{n\left(y_{\mathrm{gt}}\right)+n\left(y_{\mathrm{net}}\right)}$$

where $y_{\mathrm{net}}$ and $y_{\mathrm{gt}}$ are the predicted and the ground-truth segmentation mask, respectively, while *DSC* is the Dice similarity coefficient calculated considering the number of voxels n(·) in each volume. It is worth noting that the network expects a square-sized input. For this reason, it was used to analyse each breast separately. If, as usually happens, the dataset is bilateral (i.e., includes both breasts), a simple pre-processing is required before feeding the data to the input. More in detail, let *X* be the size of the *x* dimension according to the coordinate system introduced in Figure 3. The cutting plane x=X/2 is used to split the 3D volume in two, obtaining a separate 3D volume for each breast. These two sub-volumes are analysed separately by the network, and only the prediction will be merged to restore the original shape before providing it to the physician. A useful side-effect of this pre-processing is the doubling of training data.

### 3.5. The “Eras/Epochs” Training Schema

When performing lesion segmentation, one critical issue is the great imbalance between voxels belonging to lesioned tissue and the others. In particular, since the lesion segmentation is performed for all the slices, the data consists of *healthy slices* (with no voxels belonging to a lesion) and of *lesion slices* (in which at least one voxel belongs to a lesion). Since a lesion is usually a small portion of the whole breast, the number of *healthy* is higher than the number of *lesion* slices.

Data balancing aims to ensure that during the training phase the input of the network is a balanced dataset. In more detail, for each training step we want the network to process the same number of healthy and lesion slices. Moreover, despite the number of the network’s parameters being not particularly high (∼7.7 M for an input size of 128 × 128 pixels 3TP slices), a data-augmentation strategy and training regularization are needed to increase the network robustness.

**Figure 5:**
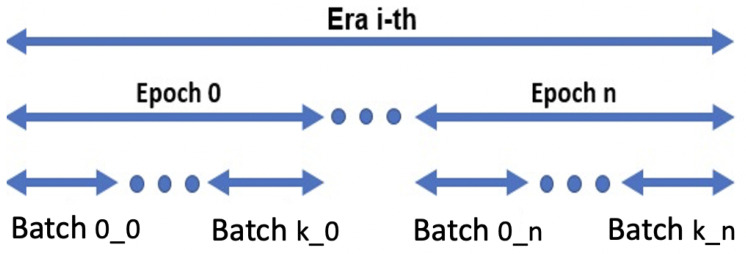
Eras/epochs training schema: there are as many eras *i* as needed by the network to converge; there are as many epochs *n* as the number of chunks; finally, within each epoch *n*, *k* batches are built by using the samples from the corresponding chunk $c_{n}$.

To address these needs, in this work, we introduce a new training schema designed to make the model train on images coming from the minority class more often than on those coming from the majority one. In practice, the “Eras/Epochs” training schema modifies the “definition” of *an epoch* from *“the network having seen all the training samples”* to *“the network having seen all the* ***minority class*** *training samples and an equivalent number of randomly chosen samples from the majority class”*. We then introduce two new terms: **“chunk”**, representing the portion of the training dataset seen during a given epoch, and **“era”**, referring to the network having seen all the samples from the majority class. From this moment, the training algorithm proceeds as a standard training schema, with the sole difference that *(i)* the batches (if applicable) are extracted within a chunk and *(ii)* that the procedure has to cycle over eras instead of over epochs (Figure 5). Summing up, the number of chunks strongly depends on the imbalance between the slices, since new chunks are created until all the *healthy slices* have been assigned to at least one chunk. As a consequence, after having processed **one** chunk, the network has processed all the *lesion slices* (i.e., a *epoch* has occurred), while after having processed **all** the chunks, the network has seen all the slices (i.e., an *era* has occurred). The result is a training schema that enforces class balancing and that supports training regularization while performing data augmentation without using fake or modified training samples (that could lead to overestimated performance).

## 4. Experimental Results

To support the results’ reproducibility and repeatability on similar datasets, we detail all the choices made, including the used dataset and the training settings. The threshold to obtain the mask from the probability output (Section 3.4) was set to 0.5. The proposed approach was implemented by using Keras (Python 3.6) with TensorFlow 2.0 as the back-end. The experiments were run on a physical server equipped with 2× Intel(R) Xeon(R) CPUs (four cores each, running at 2.13 GHz), 32 GB of DDR4 RAM, and an Nvidia Titan XP GPU (Pascal family) having 12 GB DDR5 RAM, hosted in our HPC center (SCoPE). Volume registration, breast masking, and 3TP slice extraction steps were performed in MATLAB R2018a.

One of the greatest limitations when developing a new approach for breast-cancer analysis is the lack of publicly available datasets having the full ground truths for lesions segmentation, classification, etc. This is a common problem, often resulting in the use of private datasets (as for all the deep approaches considered in Section 2). This is the case also for this study, where we used data from a private repository of women bilateral breast DCE-MRI provided by “Istituto Nazionale Tumori, Fondazione G. Pascale” of Naples consisting of 33 patients (with ages spanning in a range from 16 to 69 and an average age of 40). All the patients underwent imaging with a 1.5 T scanner (Magnetom Symphony, Siemens Medical System, Erlangen, Germany) equipped with breast coils. DCE FLASH 3D T1-weighted coronal images were acquired (TR: 9.8 ms, TE: 4.76 ms; FA: 25°; FoV 370 × 185 mm^2^; image: 256 × 128 pixels; thickness: 2 mm; Gap: 0; acquisition time: 56 s; 80 slices spanning entire breast volume). One series ($t_{0}$) was acquired before the intravenous injection of the CA and nine series ($t_{1}$–$t_{9}$) after. In particular, 0.1 mmol/kg of a positive paramagnetic contrast agent (gadolinium-diethylene-triamine penta-acetic acid, Gd-DOTA, Dotarem, Guerbet, Roissy CdG Cedex, France) was injected using an automatic system (Spectris Solaris EP MR, MEDRAD, Inc., Indianola, PA), with an injection flow rate of 2 mL/s, followed by a flush of 10 mL of saline solution at the same rate. An experienced radiologist generated the ground truth by segmenting, at a sub-pixel level, all the histopathologically proven lesions. This task was performed by exploiting the original and *subtractive* image series (where $t_{s}=t_{1}-t_{0}$) and by analysing the whole contrast-agent dynamic evolution.

All the experiments were performed using a 10-fold cross-validation (CV) to better assess the approach’s generalisation ability. In more detail, we want to highlight that it is of crucial importance to execute cross-validation in a patient-wise fashion to reliably compare the performance of different models, avoiding the use of slices form the same patient both in the training and in the evaluation phase. For this reason, for each CV repetition, eight folds were used as the training set, one as the validation set, and one as the test set. Considering the number of 3TP slices for each patient (80) and the fact that each breast is analysed separately (thus doubling the number of slices), this results in 5280 slices (406 lesions and 4874 healthy slices) divided into 4320, 480, and 480 for train, test, and validation, respectively. *By using the introduced “Eras/Epochs” training schema, during each training era, the U-Net sees a total number of 8076 slices (equally distributed in healthy and lesion)*.

To help to deal with the inter/intra patient variability, for each cross-validation fold, we performed a z-score normalization. In particular, we determined the used mean and standard deviation only on the training fold then applied them to both validation and test folds. For each cross-validation repetition, the network’s weights had been drawn from a random normal distribution $N(0,\sqrt{2/({\mathrm{fan}}_{i}+{\mathrm{fan}}_{o})})$ [45], where $fan_{i}$ and $fan_{o}$ are the input and output size of the convolution layer, respectively; the bias had been set to the constant value of 0.1. ADAM had been used as the optimiser, with $\beta_{1}=0.9$, $\beta_{2}=0.999$; the learning rate had been fixed to $10^{-4}$. The number of training eras had been fixed to 20.

To assess the performance of the proposed approach, in Table 3 we compare our results with those obtained by using some literature proposals in terms of the Dice similarity coefficient (DSC). In particular, among all the works introduced in Section 2, we compare against all deep ones and against our previous non-deep solution [22]. As a baseline, we also compare against our 3TP U-Net, in the settings and setup described in [13], and thus not as part of the pipelined approach introduced in this work. Since none of the other competitors has a publicly available implementation, we re-implemented all the approaches to the best of our understanding, following (where available) all the settings and choices made. In this regard, it is worth noting that one of the approaches [31] listed in Section 2 was not reported. The reason is that, despite our best efforts, we were not able to fully reproduce the authors’ approach, resulting in very poor results (probably also due to the strong differences in the used dataset). We want also to highlight that, in [34], the authors used a two-channel (pre-contrast and first post-contrast series) input U-Net architecture without explicitly identifying these series in terms of seconds. Considering the differences between the acquisition time in their and in our dataset, the fairer situation is to consider our pre-contrast and second post-contrast series as input to their network.

**Figure 6:**
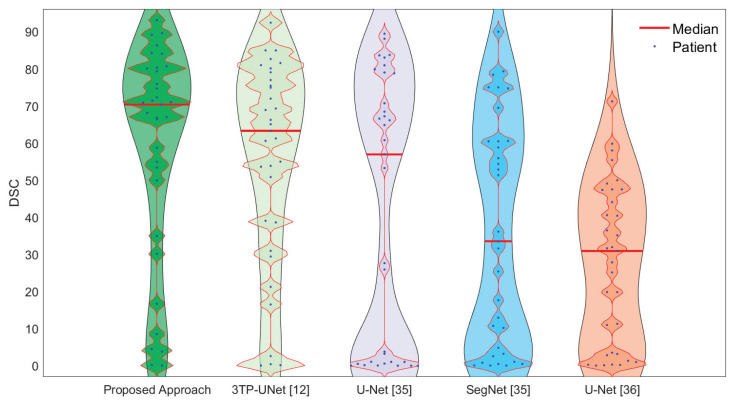
Violin plots for patient-wise (dots in the image) segmentation performance for all the deep-learning-based approaches considered in this work. For a fair comparison, all the plots were generated by setting the kernel density bandwidth to 10.

The reported results show that our approach outperformed the competitors by a large margin, with +9.13% over the runner-up. This is even more interesting considering that the second-ranked is our previous proposal [13] and that the first real competitor ranked only third, with a margin of +11.53%. To frame these numbers, it is very important to highlight that, in the respective studies, some works [31,34] measure performance by only considering the predictions made on slices actually containing a lesion. This is, in our humble opinion, not realistic (since in a clinical scenario we want the approach to analyse the whole breast looking for lesions) and unfair (as it does not take into account for *false positives*, if any, on slices different from those selected by the radiologist), resulting in performance overestimation. To sustain this claim, we evaluated the performance obtained by our model and by the one proposed in [34] considering only slices with lesions, obtaining 72.23% and 38.82% DSC values, respectively. As expected, the competitor [34] strongly benefits from this new setting (+7.90 w.r.t. Table 3), while our proposal is more resilient (+1.86 w.r.t. Table 3), highlighting a higher robustness and generalization ability. To further analyse this aspect, Figure 6 reports the violin plots for the patient-wise (dots in the image) segmentation performance of all the considered deep approaches. For a fair estimate on the real population, all the violins were generated by setting the kernel density bandwidth to 10. For each violin, dots on the same line represent different patients (for the considered dataset) having close DSC values.

The figure is rich in information, showing not only the superior performance of the proposed approach (the median values are identified by the red lines) but also its higher generalization ability. This is represented by the fact that our solution presents a single bulge in the upper part of the plot, where the biggest portion of patients (dots) are gathered, with a slimmer silhouette in the lower part. The same information, but seen from a different point of view, is reported in Table 4 where, for each technique, the corresponding DSC “ranking” is reported in terms of how often it is the 1st, the 2nd, etc. The table shows that the proposed solution is always in the top three (0% for both 4th and 5th positions), resulting to be the best solution for more than 50% of the patients (which is, in turn, more than twice the runner-up) and in the top two for ∼85% of times.

One of the key aspects of this study is the use of different stages, each intended to deal with a specific problem. We argue that all these steps contribute to the final outcome. Therefore, given that the proposed approach consists of the following five main characteristics:

- Breast-mask application for removing extraneous voxels.
- Motion correction.
- The use of 3TP slices.
- Data balancing/augmentation by using the introduced eras/epochs training schema.
- The use of a modified U-Net architecture.

we evaluated the performance of some variants (Table 5) resulting from the modification and/or deletion of some of the corresponding stages: with and without the breast-masking step (BM); with and without motion correction (MC); with and without the 3TP method, considering for the latter all the series acquired (10TP) and the use of only the pre-contrast series (1TP); with and without the use of our “eras/epochs” (EE) training schema, considering for the latter a typical data-augmentation schema consisting in random rotations; using our modified U-Net or the basic U-Net architecture [12]. Finally, we evaluated the performance by using U-Net++ [46], another variant of the standard U-Net that showed excellent results in the biomedical domain.

The results in Table 5 confirm that all the considered stages are important since each variation performed worse than the full-stages solution. It is worth noting that the result obtained by using only pre-contrast series (1TP) was notreported since, despite our best efforts, the network did not converge. Interestingly, besides this case, all the variants converged without showing overfitting in the training curves. This result is particularly interesting since it confirms the benefits of using post-contrast series in lesion segmentation.

## 5. Discussions and Conclusions

This work aimed to introduce a new approach for the automatic lesion segmentation in breast DCE-MRI, explicitly designed to leverage the physiological information associated with it. To this aim, we proposed a pipelined approach where each stage is intended to deal with or to leverage a peculiar characteristic of breast DCE-MRI data: the use of breast-masking pre-processing to remove non-breast tissues; the use of the Three-Time-Points (3TP) method [11] to effectively highlight the contrast-agent time course by generating 3TP slices; the application of a motion-correction technique to deal with patient involuntary movements during the acquisition; the leverage of a modified U-Net architecture to better fit our proposes; the introduction of a new training strategy (named “Eras/Epochs”) to handle the unbalanced dataset while performing a strong data augmentation.

To show the effectiveness of the proposed approach, we compared (Table 3) our solution against some literature approaches. The results show not only that our approach outperformed the competitors by a large margin (+11.53% over the first third-party competitor) but also that it is more stable and reliable. These last claims are sustained both by the shape of the violin plot (Figure 6) and by the smaller performance improvement that we obtained when evaluating performance only on slices actually containing a lesion (thus implying that our approach has a lower level of false positives). One of the claims we made in this study is that the effectiveness of the proposed approach lays the foundations in the use of several stages, each properly chosen to deal with a given problem. To prove this, we performed an ablation analysis (Table 5) clearly showing the impact of each stage, highlighting, among all, the importance of breast masking and of motion correction.

As described in the previous sections, having several (and often very different between different protocols) acquisitions, as in DCE-MRI, poses a problem for the use of Deep Learning. In this regard, one of the greatest advantages of our approach is that by using the 3TP method, we uniquely and clearly defined which are the acquisition series to use in terms of seconds after the CA injection. This has two main consequences: (i) we can use a network with fewer parameters than those considering all the acquisition times, resulting in a better convergence when dealing with small datasets; (ii) that our approach can be applied on all DCE-MRI protocols involving at least three acquisitions (the only constraint is the need to have acquisitions close to the times suggested in [11]). Since this latter aspect may represent a turning point towards the development of a protocol-independent Deep-Learning approach for the analysis of breast DCE-MRI data, future works will analyse how reliable are the considered time points as some external parameters (e.g., manufacturer, field strength, etc.) changes. Nonetheless, the reported results further confirms our idea that combining past learned experience in the radiomics field and Deep Learning is the right strategy to improve the effectiveness of automatic breast-cancer analysis [47].

Another crucial aspect to be taken into account is the size of the dataset used in this study. Despite the number of involved patients being relatively small, this did not affect the training procedure since, as aforementioned, the use of 3TP slices and of a modified U-Net architecture resulted in an architecture having a reduced number of parameters (and thus requiring a smaller number of training samples to converge). Moreover, the use of the introduced “Eras/Epochs” training schema allows for a simple and effective class balancing, while enforcing a strong data augmentation without the need for generating fake data (e.g., by means of rotations, scale, etc.). In particular, considering the number of 3TP slices for each patient (80) and the fact that each breast is analysed separately (thus doubling the number of slices), the use of the proposed training schema makes the network train on 8076 slices during each training era. Finally, the use of 10-fold per-patient cross-validation further sustains the reliability of the reported results.

## Figures and Tables

**Table 1:** Common breast DCE-MRI hand-crafted features for lesion segmentation, grouped by the leveraged characteristics.

Feature Class	Acronym	Characterization
Dynamics	DYN	Features quantifying the dynamic (i.e., kinetic) of the contrast agent, measured on the time intensity curve (TIC).
Textural Features	TXT	Features designed to measure the global/local perceived texture of the input image.
Geometrical	GEO	Features describing the shape and the surface of a lesion.

**Table 2:** Short list of some non-deep approaches for breast lesion segmentation. For each entry, the table reports the publication year, whether it uses a MCT, the used set of features, the approach category, and the obtained results, as reported by the authors (ACC: voxel-based accuracy; AUC: area under the ROC curve; DR: detection rate; DSC: Dice similarity coefficient; HD: voxel-based Hausdorff distance; OR: overlap ratio; SEN: voxel-based sensitivity).

Study	Year	MCT	Features	Approach	Performance
Agner et al. [14]	2009		DYN	MODEL, MORPH	HD 11.57
Bhooshan et al. [15]	2010		DYN	MODEL	AUC 0.83
Cai et al. [16]	2014		DYN	MODEL, MORPH	AUC 0.93
Dalmis et al. [17]	2016		DYN	MORPH	AUC 0.85
Fusco et al. [18]	2012		DYN, GEO	MODEL	ACC 0.91
Hassanien et al. [19]	2012		TEX	MODEL	ACC 0.98
Jayender et al. [20]	2014		DYN	MODEL	DSC 0.77
Lee et al. [21]	2010	✓	DYN	MODEL	AUC 0.88
Marrone et al. [22]	2013		DYN	MODEL	ACC 0.98
McClymont et al. [23]	2014	✓	DYN	MODEL, MORPH	DSC 0.76
Moftah et al. [24]	2014		DYN	MODEL	ACC 0.89
Nagarajan et al. [25]	2013		DYN	MODEL	AUC 0.82
Vignati et al. [26]	2009	✓	DYN	FILT	SEN 0.93
Vignati et al. [27]	2011	✓	DYN	FILT	DR 0.89
Wang et al. [28]	2013	✓	DYN	MODEL	OR 0.93
Wang et al. [29]	2014		DYN	MORPH	ACC 0.91
Zheng et al. [30]	2009		DYN	MORPH	ACC 0.97

**Table 3:** Comparison of the proposed approach against some literature competitors in terms of median DSC values over a 10-fold CV. For the sake of fairness, we also report the performance of each competitor algorithm as reported by the authors (ACC: accuracy; DSC: Dice similarity coefficient; IoU: intersection over union).

Method	Approach	DSC [%]	Performance
Our solution	Pipelined U-Net	**70.37%**	–
Piantadosi et al. [13]	3TP U-Net	61.24%	DSC 61.24%
El Adoui et al. [33]	U-Net	58.84%	IoU 76.14%
El Adoui et al. [33]	SegNet	31.60%	IoU 68.88%
Spuhler et al. [34]	U-Net	30.92%	DSC 71.00%
Marrone et al. [22]	SVM	19.07%	ACC 98.70%

**Table 4:** Analysed deep approaches DSC performance ranking in terms of how often a given solution (on the rows) resulted to be the best (the first), the runner-up (the second), and so on (on the columns).

Method	1st	2nd	3rd	4th	5th
Proposed approach	51.52%	33.33%	15.15%	0%	0%
3TP U-Net [13]	24.24%	24.24%	33.33%	18.19%	0%
U-Net [33]	21.21%	30.30%	18.18 %	12.12%	18.19%
SegNet [33]	0%	12.13 %	21.21 %	42.42%	24.24%
U-Net [34]	3.03 %	0 %	12.13%	27.27%	57.57%

**Table 5:** Variant analysis results in terms of DSC median values over a 10-fold CV. Text in bold refers to the best-performing variant. It is worth noting that the result obtained by using only pre-contrast series (1TP) has not been reported since, despite our best efforts, it did not converge.

BM	MC	TP	EE	Model	DSC
YES	YES	3TP	YES	Our U-Net	**70.37%**
NO	YES	3TP	YES	Our U-Net	53.35%
YES	NO	3TP	YES	Our U-Net	59.84%
YES	YES	10TP	YES	Our U-Net	67.26%
YES	YES	3TP	NO	Our U-Net	68.90%
YES	YES	3TP	YES	Basic U-Net	67.17%
YES	YES	3TP	YES	U-Net++	65.12%

## Data Availability

Restrictions apply to the availability of these data. Data was kindly provided by Antonella Petrillo, Head of Division of Radiology of “IRCCS Istituto Nazionale dei Tumori Fondazione G. Pascale” (Naples, Italy).
